# Prebiotic Iron Originates the Peptidyl Transfer Origin

**DOI:** 10.1093/molbev/msz034

**Published:** 2019-02-19

**Authors:** Shin-Yi Lin, Ying-Chi Wang, Chiaolong Hsiao

**Affiliations:** Institute of Biochemical Sciences, National Taiwan University, Taipei, Taiwan

**Keywords:** ribosomal evolution, electron transfer, ribosomal RNA, Fe-microcluster, peptidyl transfer origin

## Abstract

The ribosome is responsible for protein synthesis in all living organisms. It is best known to exist around 3.5–3.7 Ga whereat life on Earth inhabited anoxic environment with abundant soluble irons. The RNAs and proteins are the two biopolymers that constitute the ribosome. However, both proteins and RNAs require metal cations to fold and to function. There are four Mg-microcluster (Mg^2+^-μc) structures conserved in core of large subunit, and the 23S ribosomal RNA (rRNA) was shown to catalyze electron transfer in an anoxic environment in the presence of Fe^2+^. The Mg^2+^-μc features two idiosyncratic Mg^2+^ ions that are chelated and bridged by a common phosphate group and along with that, the adjacent residues of RNA backbone together forming ten-membered chelation ring(s). Here, we utilized four rRNA fragments of the large subunit 23S rRNA of *Haloarcula marismortui*, that includes the residues that form the four Mg^2+^-μc’s. These four rRNA fragments are shown competent to assemble with Mg^2+^. Our results show that when these rRNA fragments fold or assembly in the presence of Fe^2+^ under anoxic conditions, each Fe^2+^-microcluster can catalyze electron transfer. We propose that Fe^2+^-microclusters of the ribosome, which use Fe^2+^ as a cofactor to regulate electron transfer, are pivotal and primordial and may be an origin in evolution of the ribosome.

## Introduction

The ribosome is the operating system (Bowman et al. 2015) for protein synthesis in all living organisms. It is best known to exist around 3.5–3.7 Ga whereat life on Earth inhabited anoxic environment with abundant soluble irons (Schopf et al. 2002; Anbar 2008; Ohtomo et al. 2014). The RNAs and proteins are the two biopolymers that constitute the ribosome. However, protein requires metal cations to fold and to function, so as RNA. There are four Mg-microcluster (Mg^2+^-μc) structures conserved in core of large subunit (LSU) ribosomes, where the three Mg^2+^-μc’s were proposed to provide a framework for the peptidyl transfer center (PTC) (Hsiao and Williams 2009). The Mg^2+^-μc’s (Hsiao and Williams 2009) are seen to 1) exhibit two idiosyncratic Mg^2+^ ions, 2) possess a common phosphate group that chelates and bridges the two Mg^2+^ ions, and 3) form ten-membered chelation ring(s) (supplementary fig. S1, Supplementary Material online). In addition, metals are essential constituents that confer varieties of catalytic functions on biological macromolecules (Riordan 1977). For example, the T4 DNA ligase requires Mg^2+^ ions to catalyze formation of the phosphodiester bond of two dsDNA fragments (Lehman 1974). The Class Ia ribonucleotide reductase (RNR) contains dinuclear iron clusters that are required to catalyze the reduction of ribonucleotide to deoxyribonucleotide (Cotruvo and Stubbe 2011). The L1 ribozyme ligase performs phosphoryl transfer in the presence of Mg^2+^ or Fe^2+^ ions (Athavale, Petrov, et al. 2012).

**Fig. 1.**
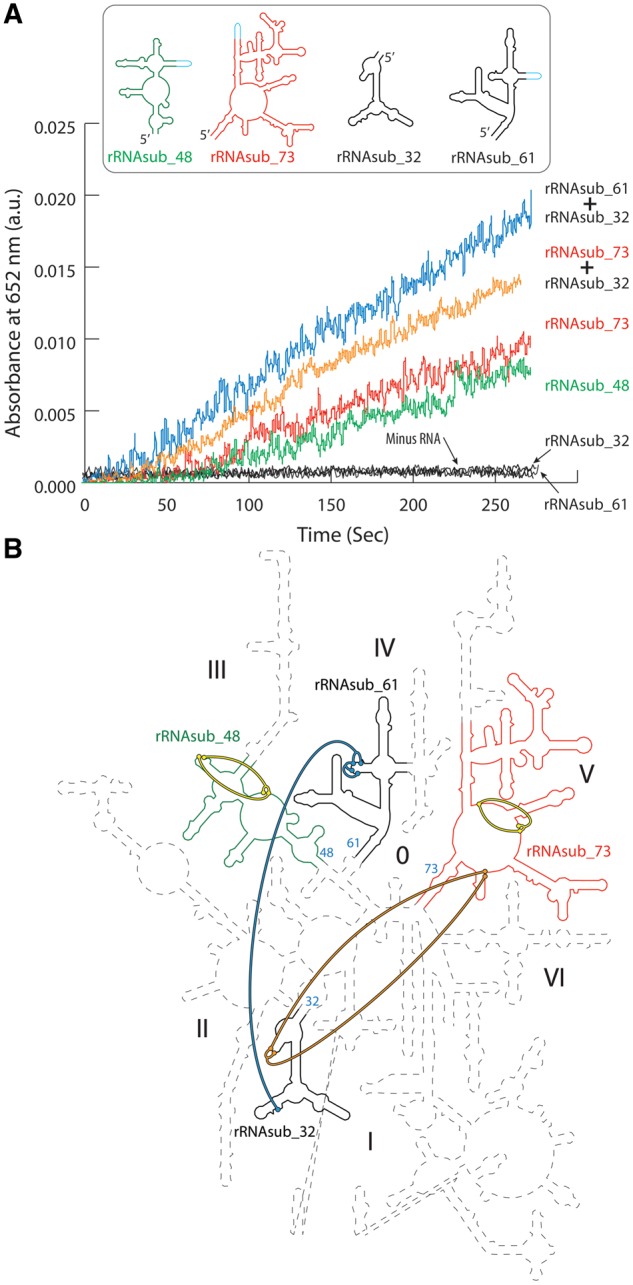
The 23S rRNAsubs and their assemblies catalyze electron transfer. (*A*) The rRNAsub_48, rRNAsub_73, the mixture of [rRNAsub_73 and rRNAsub_32], and the mixture of [rRNAsub_61 and rRNAsub_32] were observed to catalyze electron transfer in the absence of O_2_ and in the presence of Fe^2+^ and H_2_O_2_. The rRNAsub_32 and rRNAsub_61 alone by itself showed no activity. (*B*) The four rRNAsubs taken from the 23S rRNA of *Haloarcula marismortui* were highlighted on the 3D structure-based 23S rRNA secondary structure map (Petrov, Bernier, Gulen, et al. 2014). The helix number of the 23S rRNA used for nomenclature of the rRNAsubs is shown as number colored in cyan. The rRNAsub_48 is color green, the rRNAsub_73 red, and black for both the rRNAsub32 and rRNAsub_61. The four Mg^2+^-μc’s within the rRNAsubs of the ribosome (Hsiao and Williams 2009) were colored by the thick lines that represent the molecular interactions of the phosphate oxygen and the direct contact of the two Mg^2+^. The rRNAsub_48 can form one Mg^2+^-μc, shown by the yellow thick lines. The rRNAsub_61 and rRNAsub_32 together assemble one Mg^2+^-μc, shown by the blue thick lines. The rRNAsub_73 by itself has one Mg^2+^-μc (the yellow thick lines) and forms the second Mg^2+^-μc upon the assembly of the rRNAsub_32 (the orange thick lines). The thick line color schemes represented here are corresponding to the single-electron activities in (*A*).

**Fig. 2.**
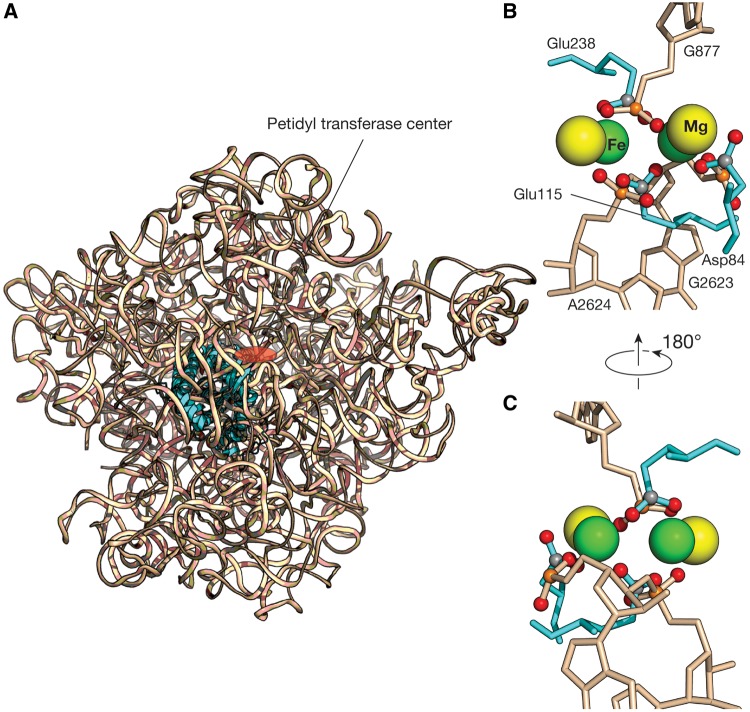
The superimposition of the Mg^2+^-μc and the dinuclear iron clusters. (*A*) One of the Mg^2+^-μc’s (Hsiao and Williams 2009) that is closest to the PTC (red circle) of the *Haloarcula marismortui* 23S rRNA (wheat ribbon, PDB entry 1JJ2) was superimposed to the catalytic center of the *Escherichia coli* RNR R2 (cyan ribbon, PDB entry 1PIY). (*B*) A close-up view of the catalytic site of this superimposition shows the di-Fe^2+^ geometry in RNR R2 is well conserved in Mg^2+^-μc of the 23S rRNA. The three carboxylic groups of the amino acid residues that are directly contacted by the Fe^2+^ are mimicked by the phosphate groups of the nucleotides that are direct contact with the Mg^2+^. (*C*) Another view of this superimposition. Green spheres denote Fe^2+^, yellow: Mg^2+^, orange: phosphorous, red: oxygen, and gray: carbon. RNA is colored wheat, and protein colored cyan.

Previous work has shown that the substitution of iron for magnesium confers the 23S ribosomal RNA (rRNA) with a new catalytic function: single-electron transfer (Hsiao, Chou, et al. 2013). Here, we report that each of the four Mg^2+^-μc’s of the 23S rRNA when substituted with Fe^2+^ demonstrates electron transfer activity (fig. 1*A*). The structure-based comparisons of these Mg^2+^-μc’s within the 23S rRNA and the dinuclear metal centers in the Class I RNRs revealed that the geometries of the two are closely related. For example, we superimpose one of the Mg^2+^-μc’s that is closest to the PTC of the *Haloarcula marismortui* LSU (PDB entry 1JJ2) with the dinuclear Fe^2+^ cluster, the catalytic center of the *Escherichia coli* RNR R2 (PDB entry 1PIY) (fig. 2*A*). The superimposition shows that the three carboxylic groups of the amino acid residues (Glu238, Asp84, and Glu115) that are directly contacted by the Fe^2+^ within the RNR are mimicked by the phosphate groups of the nucleotides (G877, G2623, and A2624) that are in direct contact with the Mg^2+^ in the 23S rRNA (fig. 2*B* and *C*). Likewise, the bridging phosphate of the residue G877 corresponds to the bridging carboxylate of the amino acid residue Glu238. In analogy to the dinuclear metal centers conferring the RNRs catalytic reduction activities, we hypothesize that one or more of the microcluster structures in the primordial 23S rRNA conferred it with the catalytic capability of electron transfer when iron (II) was abundantly available and could take the place of Mg^2+^ in the anoxic environment of early earth.

To test our hypothesis, we used the high-resolution crystallographic data of the ribosomes (Klein et al. 2001; Selmer et al. 2006) to guide and dissect the 23S rRNA into fragments that form the direct contacts with the four Mg^2+^-μc’s (fig. 1*B*). Four 23S rRNA fragments were in vitro transcribed from DNA templates (supplementary table S1, Supplementary Material online). They are referred to here as the 23S rRNA subelements (rRNAsubs) which are rRNAsub_32, rRNAsub_48, rRNAsub_61, and rRNAsub_73. The number of each rRNAsub indicates the helix number of the 23S rRNA that comprises the terminal helical stem of the 23S rRNA fragment (fig. 1*B*). These 23S rRNA fragments are different in lengths and proven an induction of folding with Mg^2+^ (supplementary fig. S2, Supplementary Material online). They provided a test of the hypothesis that one or more of the four Mg^2+^-μc’s responsible for the electron transfer activity of the 23S rRNA in association with Fe^2+^.

## Results

Two of the four rRNA fragments (rRNAsub_48 and rRNAsub_73) dissected from the 23S rRNA were found to catalyze electron transfer in the presence of Fe^2+^ (fig. 1*A*). The other two (rRNAsub_32 and rRNAsub_61) showed no enzymatic activity alone, however, the catalysis was observed upon their assembly (fig. 1*A*). This outcome is consistent with the prediction based on the 23S rRNA structure that each Mg^2+^-μc (Hsiao and Williams 2009), with Fe^2+^ substituting for Mg^2+^, is capable of electron transfer (fig. 1*B*).

**Fig. 3.**
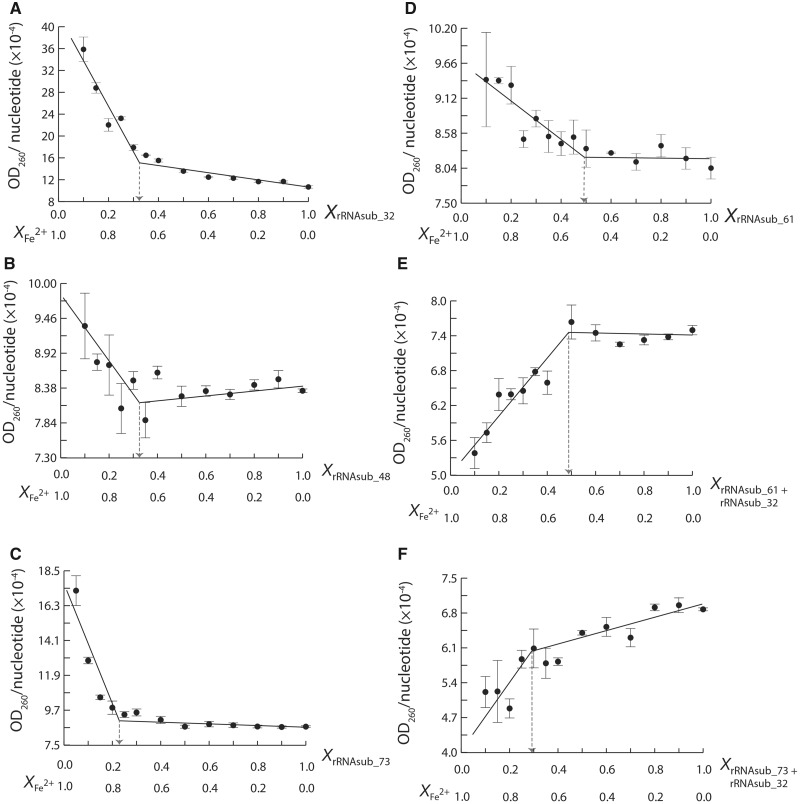
The continuous variation analysis on Fe^2+^ with (*A*) rRNAsub_32, (*B*) rRNAsub_48, (*C*) rRNAsub_73, (*D*) rRNAsub_61, (*E*) a mixture of [rRNAsub_61 and rRNAsub_32], and (*F*) a mixture of [rRNAsub_73 and rRNAsub_32]. In the plots, values on horizontal axis denote mole fraction of the rRNAsub and Fe^2+^ in each sample. The discontinuity at equivalent mole fractions of rRNAsub and Fe^2+^ indicates a complex with ratio of stoichiometry.

Continuous variation analysis (Job 1928; Cantor and Schimmel 1984) was carried out on the complexation of Fe^2+^ and the rRNAsubs in anoxic conditions to determine the stoichiometries of the Fe^2+^-rRNA complexes (fig. 3). The continuous variation of rRNAsub_32 with Fe^2+^ gave a stoichiometric 1:2 ratio of rRNAsub_32 and Fe^2+^ complexes (fig. 3*A*). A similar result was obtained in the continuous variation experiment of rRNAsub_48 with Fe^2+^ (fig. 3*B*). Iron (II) confers the rRNAsub_48 with electron transfer activity, however this was not the case for rRNAsub_32 (fig. 1*A*), although both form a stoichiometric 1:2 ratio of rRNA and Fe^2+^ complex. The continuous variation of rRNAsub_73 with Fe^2+^ formed a rRNA-Fe^2+^ complex with 1:3 stoichiometry (fig. 3*C*). This complex is capable of catalyzing electron transfer (fig. 1*A*). The continuous variation of rRNAsub_61 with Fe^2+^ forms a rRNA-Fe^2+^ complex with 1:1 stoichiometry (fig. 3*D*) and showed no electron transfer activity (fig. 1*A*).

In order to gain better understanding of the stoichiometries found for Fe^2+^ and the individual rRNAsub fragments, continuous variation experiments were employed to examine the interaction of rRNAsub_32 with rRNAsub_61 and rRNAsub_32 with rRNAsub_73, and the stoichiometry of these complexes with Fe^2+^. Two of the Mg^2+^-μc’s of the 23S rRNA are formed from these pairs of rRNAsub fragments (fig. 1*B*). When we mixed the rRNAsub_32 (of domain II) with rRNAsub_61 (of domain IV) in the presence of Fe^2+^ in a continuous variation experiment, the results showed a formation of a specific RNA–RNA complex with 1:1 stoichiometry (supplementary fig. S3*A*, Supplementary Material online). The same 1:1 stoichiometric complex was observed for rRNAsub_32 (of domain II) and rRNAsub_73 (of domain V) in the presence of Fe^2+^ (supplementary fig. S3*B*, Supplementary Material online). Moreover, the continuous variation of the mixture [rRNAsub_32 and rRNAsub_61] with Fe^2+^ showed a rRNA-Fe^2+^ assembly with a 2:2 stoichiometry (fig. 3*E*). An assembly with a 2:5 ratio was observed for the mixture of [rRNAsub_32 and rRNAsub_73] and Fe^2+^ (fig. 3*F*).

**Fig. 4.**
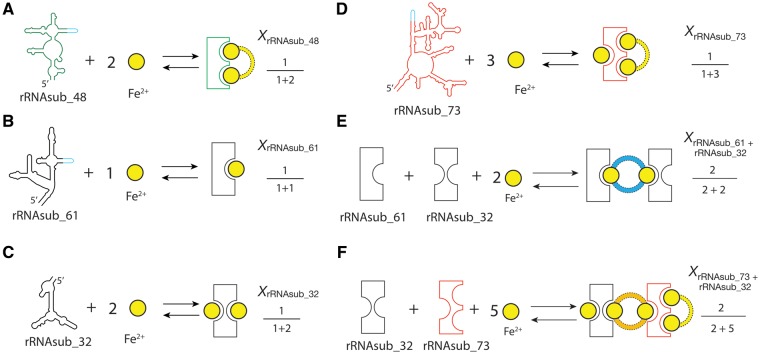
The Fe^2+^-μc of the Fe^2+^-rRNAsub complexes. Shown is the schematic presentation of the dinuclear metal binding sites of iron in (*A*) rRNAsub_48, (*B*) rRNAsub_61, (*C*) rRNAsub_32, (*D*) rRNAsub_73, (*E*) [rRNAsub_61 + rRNAsub_32], and (*F*) [rRNAsub_73 + rRNAsub_32]. The mole fractions obtained from continuous variation experiments were listed aside. The yellow circle denotes Fe^2+^ cation. The color dashed line indicates the RNA-Fe^2+^ interactions that form the Fe^2+^-μc: yellow in the rRNAsub_48 and rRNAsub_73, blue in the [rRNAsub_61 + rRNAsub_32], and orange in the [rRNAsub_73 + rRNAsub_32]; the color schemes represented here are the same, corresponding to figure 1*B*.

The electron transfer assay, the continuous variation analysis, and the Fe^2+^ dependence of the reaction rate experiments (figs. 1*A* and 3 and supplementary fig. S4, Supplementary Material online), all together revealed a Fe^2+^-μc of the Fe^2+^-rRNAsub complexes (fig. 4), indicating that there are dinuclear metal binding sites specific for Fe^2+^. Our data showed that there is one particular iron binding site in the rRNAsub_61, two in the rRNAsub_32, and three in the rRNAsub_73. The iron dependence experiment (supplementary fig. S4, Supplementary Material online), combined with continuous variation analysis (fig. 3), deduced that two idiosyncratic Fe^2+^ cations bound to the [rRNAsub_61 + rRNAsub_32] formed a Fe^2+^-μc is 56% versus an auxiliary Fe^2+^ continuously bound to that complex is 44% (supplementary fig. S5*A*, Supplementary Material online). In the case of [rRNAsub_73 + rRNAsub_32], a pair of two idiosyncratic Fe^2+^ cations were assembled into two Fe^2+^-μc’s are 32% versus an auxiliary Fe^2+^ continuously bound to that complex is 68% (supplementary fig. S5*B*, Supplementary Material online).

**Fig. 5.**
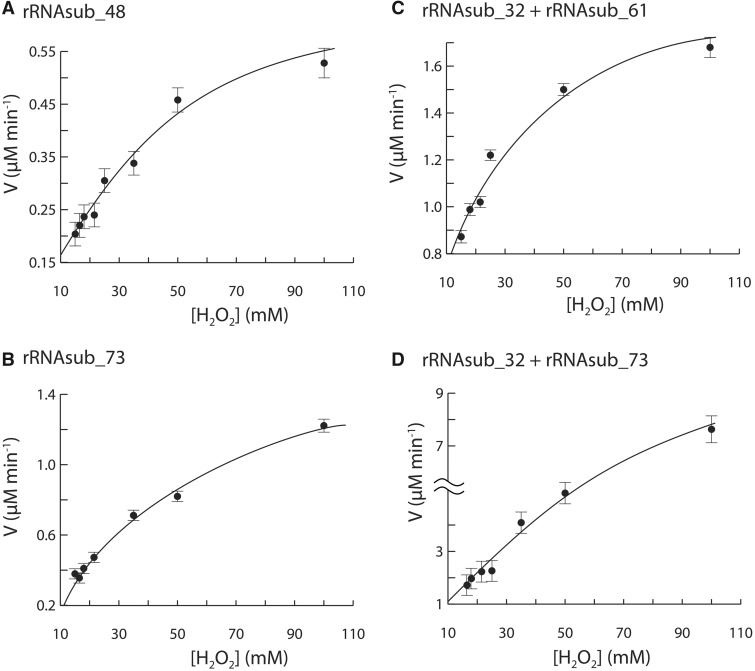
The dinuclear metal entity of the rRNAsubs with Fe^2+^ catalyzed single-electron transfer follows enzyme kinetics. The electron transfer catalyzed by (*A*) rRNAsub_48, (*B*) rRNAsub_73, (*C*) a mixture of [rRNAsub_32 and rRNAsub_61], and (*D*) a mixture of [rRNAsub_32 and rRNAsub_73] were all saturable, reaching plateau at high concentration of H_2_O_2_.

The kinetics of electron transfer catalyzed by Fe^2+^-μc’s follows the Michaelis–Menten kinetics (fig. 5). Kinetic parameters for electron transfer by rRNAsub_48, rRNAsub_73, [rRNAsub_32 and rRNAsub_61] assembly and [rRNAsub_32 and rRNAsub_73] assembly were obtained by nonlinear regression curve experimental data fitting into Michaelis–Menten models (fig. 5). For rRNAsub_48, *k*_cat_ = 0.76 min^−1^, *K*_M_ = 40.32 mM, and *k*_cat_/*K*_M_ = 0.31 M^−1^S^−1^. For rRNAsub_73, *k*_cat_ = 2.13 min^−1^, *K*_M_ = 75.02 mM, and *k*_cat_/*K*_M_ = 0.47 M^−1^S^−1^. For [rRNAsub_61 + rRNAsub_32], *k*_cat_ = 2.03 min^−1^, *K*_M_ = 19.03 mM, and *k*_cat_/*K*_M_ = 1.78 M^−1^S^−1^. For [rRNAsub_73 + rRNAsub_32], *k*_cat_ = 19.96 min^−1^, *K*_M_ = 156.57 mM, and *k*_cat_/*K*_M_ = 2.12 M^−1^S^−1^.

## Discussion

Metal cations play critical roles in all biological macromolecules, including proteins, DNAs, and RNAs. The coordination chemistry and electrostatic forces of cations have conformational effects on folding and assembly for these biological macromolecules (Brown 1988; Williamson et al. 1989; Celander and Cech 1991; Auffinger et al. 2011; Cotruvo and Stubbe 2011). Each cation exhibits specific charge density defined by the ionic radius and its charge. Small atomic radius and high charge density such as Fe^2+^ and Mg^2+^ in dinuclear metal clusters are pivotal in enzyme catalysis (Cotruvo and Stubbe 2011) and RNA folding (Hsiao et al. 2008; Hsiao and Williams 2009). For example, di-Fe metal clusters are critical for enzymatic activity in the Class Ia of RNRs, and di-Mn metal clusters are a catalytic center in the Class Ib. In RNAs, Mg^2+^-μc’s are a recurrent RNA-metal binding motif, observed in the 23S rRNAs, 16S rRNA, and the *tetrahymena* Group I intron P4-P6 ribozyme (Hsiao and Williams 2009). Four Mg^2+^-μc’s are seen in the bacterial, archaeal, and eukaryal ribosomes, which allows one to trace back life to the last universal common ancestor by several billions of years ago.

### A Ribosomal Switch On and Off Electron Transfer

It is now known that substitution for Mg^2+^ with Fe^2+^ confers the 23S rRNA an electron transfer function (Hsiao, Chou, et al. 2013). As of the RNR, the dinuclear Fe or Mn clusters are essential entities for the catalytic reduction of ribonucleotides (Cotruvo and Stubbe 2011). When we compare the coordination geometries of the catalytic centers in the Class Ia RNR and one of the Mg^2+^-μc’s in the 23S rRNA, surprisingly, they share an almost identical metal–ligand conformation, despite the two biological macromolecules are totally distinct (fig. 2). Our rRNAsub fragments folding and assembly experiments with Fe^2+^ reveal a Fe^2+^-μc that remarks the electron transfer activity. It is when Fe^2+^ substitutes the two idiosyncratic Mg^2+^ cations within the Mg^2+^-μc conferring the 23S rRNA a capability of electron transfer function. When rRNAsub_32 is alone in the presence of Fe^2+^, there was no catalytic activity in despite a 1:2 stoichiometric rRNA-Fe^2+^complexes were observed (figs. 1*A*, 3*A*, and 4*C*), suggesting that the Fe^2+^-μc was not formed. Notably, this catalytic activity can be summoned by the addition of rRNAsub_61, upon with their assembly (figs. 1*A*, 3*E*, and 4*E*). Thus, the Fe^2+^-μc of the 23S rRNA is a ribosomal switch that can turn on and off the electron transfer.

### Fe^2+^-μc May Be an Origin in Evolution of the Ribosome

There are four Mg^2+^-μc’s in LSU of the ribosome, conserved in sequences and structures over three domains of life. One of the Mg^2+^-μc’s formed a portion of the peptidyl exit tunnel was suggested to be added late in ribosomal evolution (Athavale, Gossett, et al. 2012). The other three are close to the PTC that provides a framework for ribosomal peptidyl transfer (PT) (Hsiao and Williams 2009). The PTC is the catalytic site of the ribosome that catalyzes the peptide formation. It consists of two terminal loops, the A and P loops that position the aminoacyl-tRNA and peptidyl-tRNA in an appropriate conformation to catalytically facilitate the formation of peptide bond in an entropic manner (Sievers et al. 2004). Structural analysis of the PTCs in 2D and 3D structures reveal a symmetric pocketlike assembly. This pocketlike assembly is a pseudo 2-fold symmetric entity, named proto-ribosome (Agmon 2009; Belousoff et al. 2010). Further structural investigations on models of the proto-ribosomes, we observed that there is one conserved Mg^2+^-μc that is intimate bridging the P- and A-regions of the 23S rRNA. The sequence contents of the proto-ribosomes are highly conserved in all organisms across the phylogenic tree. In addition, the conformations and geometries of the local structure formed by the Mg^2+^-μc and the 23S rRNA are very well conserved. As of the translation is believed to appear originally not to synthesize proteins, but allow small peptides bound to small RNAs such that to extend RNA’s structural capabilities (Fahnestock et al. 1970; Noller 2004). When we examined the 2D structures of the proto-ribosome and our four rRNA fragments, the proto-ribosome is strikingly similar to the rRNAsub_73. Comparisons of ribosomal evolution on rRNAsub_73 indicate that it might be the most ancient part of the ribosome goes beyond last universal common ancestor (Bokov and Steinberg 2009; Hsiao et al. 2009; Fox 2010; Petrov et al. 2015).

In models of ribosomal evolution, rRNA fragments were “elements” to be added as layers on top of the PT origin throughout its history (Bokov and Steinberg 2009; Hsiao et al. 2009; Petrov, Bernier, Hsiao, et al. 2014). Likewise, Fe^2+^ and Mn^2+^ are suggested to play a role in early ribosomal evolution. Bray et al. (2018) demonstrated that substitution for Mg^2+^ with Fe^2+^ and Mn^2+^ as cofactors in ribosomes can mediate in vitro protein production. Their work supports the translation machinery matured when ancient Earth conditions were anoxic and abundant with soluble Fe^2+^ and Mn^2+^ (Schopf et al. 2002; Anbar 2008; Johnson et al. 2016). Our study here shows that rRNAsub_73 formed a Fe^2+^-μc in the presence of Fe^2+^ is capable of catalyzing electron transfer. Thus, it is possible that Fe^2+^-μc complexes with a very ancient rRNA fragment such as rRNAsub_73 to form a PT origin and act as a nucleation for evolution of the translation just like a proto-ribosome proposed by Yonath and coworkers (Belousoff et al. 2010; Krupkin et al. 2011). However, the PT origin might have originated in electron transfer rather than PT. Interestingly, rRNAsub_73, rRNAsub_48, [rRNAsub_61 and rRNAsub_32], and [rRNAsub_73 and rRNAsub_32] failed of catalyzing electron transfer in the presence of Mn^2+^, and in the absence of Fe^2+^ and O_2_ (data not shown) suggests that electron transfer might be, in part, a selection pressure in Fe^2+^-μc that originated the PT origin. Additionally, we observed the initial rate of reaction for each Fe^2+^-μc increased (ca. 1- to 9-folds, see supplementary table S2, Supplementary Material online) when Fe^2+^ and Mn^2+^ were both present in the reaction, which implies that prebiotic Mn^2+^ may play a role as an ancillary element that stabilizes the Fe^2+^-μc structures in early ribosomal evolution.

In this study, rRNAsub_32 is recruited and assembled with rRNAsub_73 and formed a Fe^2+^-μc (fig. 1*B*), where we think that rRNAsub_61 fragment might be an evolutionary selection through the formation of Fe^2+^-μc with rRNasub_32 and was added after the assembly of [rRNAsub_73 and rRNAsub_32] complex. Thus, our results suggest that rRNAsub_73, rRNAsub_32, and rRNAsub_61 together formed a complex with three Fe^2+^-μc’s embedded may be a core of LSU in early ribosomal evolution. These three rRNA fragments are proposed as the essential elements of core of LSU (Fox 2010; Hsiao, Lenz, et al. 2013; Petrov et al. 2015). We show that Fe^2+^-μc of the ribosome is pivotal and primordial and may be an origin in evolution of the ribosome.

## Materials and Methods

### Design of the 23S rRNAsub Genes

The rRNAsubs were designed to include residues that have direct first-shell contact of the di-Mg^2+^ (Hsiao and Williams 2009), guided by dissecting the high-resolution crystallographic data of the LSU of the *H. marismortui* ribosome (PDB entry 1JJ2). The dissected 23S rRNA fragments were categorized by its domain. This dissecting process left two fragments in each domain III, IV, and V (fig. 1*B*). The two fragments were then joined by a tetraloop with a sequence (5′-GGGGTAACCC-3′). Therefore, the four rRNAsub’s genes were obtainable and commercially purchased from *Omics Bio*. In addition, each rRNAsub gene was added a T7 promoter at the 5′-teminus and the two restriction sites, *Hin*dIII at 5′- and *Bam*HI at 3′-end. The gene sequence of the rRNAsubs was listed in the supplementary table S1, Supplementary Material online.

### Synthesis and Purification of the rRNAsubs

The commercially purchased rRNAsub genes from *Omics Bio* were already cloned in pUC57 vector. We transformed the plasmid containing the rRNAsub gene into DH5α competent cells by heat shock technique and incubated at 37 °C for 16 h prior to plating to Luria-Bertani (LB) agar containing 100 µg/ml ampicillin. Colonies positive for insert were used to inoculate 3 ml of LB containing 100 µg/ml ampicillin. After 16 h of incubation at 37 °C, plasmids were purified by QIAPrep Spin Miniprep Kit (Qiagen).

*The in vitro transcripts*. All rRNAsubs were prepared and synthesized by using MEGAscript transcription kit (Ambion) to avoid any in vivo contaminations for the experiments. To synthesize the in vitro rRNAsubs, 1 µg of linearized template DNA with a T7 promoter was mixed with NTPs, 10× reaction buffer, enzyme mix and nuclease-free water to make the total reaction volume 20 µl, and incubated at 37 °C. The optimum in vitro transcription time for each rRNAsub production is as follows: 7 h for rRNAsub_32, 6 h for rRNAsub_48, 6 h for rRNAsub_61, and 4 h for rRNAsub_73. After in vitro transcription reaction, the transcripts were precipitated by adding equal volume of a fresh prepared 5-M ammonium acetate solution, and placed it on ice for 15 min, followed by centrifuged at speed 10,000 × g, at 4 °C for 15 min. The RNA pallet was then washed twice by using 80% ethanol, lyophilized, and resuspended in 50-µl nuclease-free water.

### The Single-Electron Transfer Assay

The in vitro transcripts used in the experiments were first mixed with the cation exchange resin (Chelex 100 Resin, Bio-RAD) and incubated at 60 °C for 1 min. The RNAs were then recovered by using a 0.22-μm Ultrafree-MC Centricon (Millipore), centrifuged at 10,000 × g to remove resin. The resulting RNA solution was then lyophilized for later use. The single-electron transfer assay was described detail in Hsiao, Chou, et al. (2013). The resin-treated rRNAsubs were resuspend in 20-mM 4-(2-hydroxyethyl)-1-piperazineethanesulfonic acid (HEPES)–Tris buffer, pH 7, and mixed with 500-μM 3,3′,5,5′-tetramethylbenzidine in Dimethyl sulfoxide (DMSO) and 50-mM H_2_O_2_ solution. The mixtures were sealed in a 1.5-ml centrifuged tube and deoxygenated by bubbling argon for 5 min. After deoxygenated, a 20-mM FeSO_4_ solution was added to the RNA containing solution to the final Fe^2+^ concentration of 100 μM and incubated at room temperature for 5 min followed by the addition of 200-mM deoxygenated H_2_SO_4_ solution to pH 6.1. The reaction was then monitored the UV–Vis absorption at 652 nm by using Ocean optics USB2000+ spectrophotometer. The data readings were recorded by using the commercial software SpectraSuite ver. 1.0 (Ocean Optics). The rRNAsub final concentrations used in each test were 0.1 μM for every rRNAsub and their assemblies.

### The Iron Dependency Analysis

The electron transfer experiments were described above and performed by varying the Fe^2+^ concentrations at 8–200 μM. The initial rate of each electron transfer at different Fe^2+^ concentration was determined by fitting linear regression to data. The iron dependency is then plotted natural logarithm of the initial rate of electron transfer versus natural logarithm of Fe^2+^ concentration (supplementary fig. S5, Supplementary Material online).

### The Continuous Variation Assay

A deionized, nuclease-free water was first deoxygenated by bubbling argon. The deoxygenated water was then transferred into the anaerobic chamber (Coy). All chemical powders used in the experiments were deoxygenated prior to transferring into the chamber. The reaction buffer 100-mM Tris-Cl, pH 8 and the 10 mM FeSO4 solution were freshly prepared inside the chamber. The 10-mM MgCl_2_ solution was prepared outside the chamber. All the RNAs used in the experiments were mixed with the cation exchange resin (Chelex 100 Resin, Bio-RAD) and incubated at 60 °C for 1 min. The RNAs were then recovered by using a 0.22μm Ultrafree-MC Centricon (Millipore), lyophilized, and then transferred into anaerobic chamber for later use. The continuous variation experiments with Mg^2+^ were performed in an aerobic environment.

#### The continuous variation analysis on the rRNAsubs with Fe^2+^/Mg^2+^

The rRNAsub and Fe^2+^/Mg^2+^ were mixed and held constant at 0.4 μM, whereas the mole fractions of the two components were varied from 0.0 to 1.0. For the rRNAsub assemblies (e.g., rRNAsub_73 + rRNAsub_32) with Fe^2+^/Mg^2+^, the two components were mixed and held constant at 0.8 μM. The mixtures were prepared in 50 mM Tris-Cl buffer, pH 8, incubated at 25 °C for 15 min and then stored at 4 °C. The UV–Vis absorption of the RNA-Fe^2+^ mixtures were measured at 260 nm at 25 °C using a Nanodrop (Thermo) inside the anaerobic chamber, and the RNA-Mg^2+^ mixtures were measured outside the chamber.

#### The continuous variation analysis on the rRNAsubs in the presence of Fe^2+^/Mg^2+^

The study of the two rRNAsubs were mixed and held constant at 0.2 μM, whereas the mole fractions of the two components were varied from 0.0 to 1.0. The RNA mixtures were prepared in 50-mM Tris-Cl buffer, pH 8 and 32-μM Fe^2+^ solution, incubated at 25 °C for 20 min and then stored at 4 °C. The RNA-Mg^2+^ mixtures were prepared in 10 mM Mg^2+^ and 100 mM Na^+^, incubated at 25 °C for 20 min and then stored at 4 °C. The UV–Vis absorption of the rRNAsubs interactions in the presence of Fe^2+^ were measured at 260 nm at 25 °C using a Nanodrop inside the anaerobic chamber, and the mixture samples were measured in an aerobic environment for the samples in the presence of Mg^2+^.

#### The continuous variation experiment data analysis

The UV–Vis absorption of each mixture sample was measured repetitively five times. Every experiment was performed twice. The measured outliers of each data set were excluded using the cutoff of 1.5 standard deviation (1.5*σ*). After excluded the outliers, the experimental data were then averaged and expressed in OD_260_ per nucleotide. The discontinuity at equivalent mole fractions was then determined by solving the intersection of the two linear regression lines.

### Michaelis–Menten Kinetics

The resin-treated rRNAsubs were resuspended in 20-mM HEPES–Tris buffer, pH 7, and mixed with 500-μM 3,3′,5,5′-tetramethylbenzidine (in DMSO) and different concentrations of H_2_O_2_ solution at 15, 16.5, 18, 21.5, 25, 50, and 100 μM. The mixture samples were deoxygenated by bubbling Argon for 5 min followed by the addition of 20-mM Fe_2_SO_4_ solution to the final Fe^2+^ concentration that is optimized for the kinetics experiment of each RNA: 100 μM Fe^2+^ for rRNAsub_48, 64 μM Fe^2+^ for rRNAsub_73, 32 μM Fe^2+^ for [rRNAsub_61 + rRNAsub_32], and 32 μM Fe^2+^ for [rRNAsub_73 + rRNAsub_32] and incubated at room temperature for 5 min. The UV–Vis absorption of each electron transfer reaction was measured at 652 nm after the addition of 200-mM deoxygenated H_2_SO_4_ solution to pH 6.1. The enzymatic kinetic parameters performed by the rRNAsubs and its assemblies can then be obtained by nonlinear regression curve fitting to the experimental data using the Michaelis–Menten model.

#### Michaelis–Menten data analysis

The *V*_max_ and *K*_m_ obtained by nonlinear regression fit to the kinetics experimental data were performed by using JMP Pro 13.1 (SAS Institute Inc.) with the Michaelis–Menten formalism.

### The Gel Mobility of rRNAsub Folding with Mg^2+^

The RNA samples were treated with cation exchange resin (Chelex 100 Resin, Bio-RAD) described as above. The study on the rRNAsub folding with varied Mg^2+^ concentration: 0, 25, 50, 100, 250, 500, 1,000, 5,000 μM, 200 ng of the resin-treated RNAs were mixed with Mg^2+^ solution, heated at 90 °C for 30 s and annealed by cooling to 25 °C at a linear rate of 4 °C/min. The annealed samples were then analyzed on a native-Polyacrylamide gel electrophoresis gel.

## Supplementary Material

Supplementary Data

## References

[msz034-B1] Agmon I. 2009 The dimeric proto-ribosome: structural details and possible implications on the origin of life. Int J Mol Sci. 10(7): 2921–2934. 19742176 10.3390/ijms10072921 PMC2738903

[msz034-B2] Anbar AD. 2008 Oceans. Elements and evolution. Science 322(5907): 1481–1483. 19056967 10.1126/science.1163100

[msz034-B3] Athavale SS, Gossett JJ, Hsiao C, Bowman JC, O’Neill E, Hershkovitz E, Preeprem T, Hud NV, Wartell RM, Harvey SC, et al 2012 Domain III of the *T. thermophilus* 23S rRNA folds independently to a near-native state. RNA 18(4): 752–758. 22334759 10.1261/rna.030692.111 PMC3312562

[msz034-B4] Athavale SS, Petrov AS, Hsiao C, Watkins D, Prickett CD, Gossett JJ, Lie L, Bowman JC, O’Neill E, Bernier CR, et al 2012 RNA folding and catalysis mediated by iron (II). PLoS One 7(5): e38024. 22701543 10.1371/journal.pone.0038024 PMC3365117

[msz034-B5] Auffinger P, Grover N, Westhof E. 2011 Metal ion binding to RNA. Met Ions Life Sci. 9:1–35. 22010267

[msz034-B6] Belousoff MJ, Davidovich C, Zimmerman E, Caspi Y, Wekselman I, Rozenszajn L, Shapira T, Sade-Falk O, Taha L, Bashan A, et al 2010 Ancient machinery embedded in the contemporary ribosome. Biochem Soc Trans. 38(2): 422–427. 20298195 10.1042/BST0380422

[msz034-B7] Bokov K, Steinberg SV. 2009 A hierarchical model for evolution of 23S ribosomal RNA. Nature 457(7232): 977–980. 19225518 10.1038/nature07749

[msz034-B8] Bowman JC, Hud NV, Williams LD. 2015 The ribosome challenge to the RNA world. J Mol Evol. 80(3–4): 143–161. 25739364 10.1007/s00239-015-9669-9

[msz034-B9] Bray MS, Lenz TK, Haynes JW, Bowman JC, Petrov AS, Reddi AR, Hud NV, Williams LD, Glass JB. 2018 Multiple prebiotic metals mediate translation. Proc Natl Acad Sci U S A. 115(48): 12164. 30413624 10.1073/pnas.1803636115 PMC6275528

[msz034-B10] Brown ID. 1988 What factors determine cation coordination numbers. Acta Crystallogr Sect B 44(6): 545–553.

[msz034-B11] Cantor C, Schimmel P. 1984 Biophysical chemistry (I–III). New York: Academic Press.

[msz034-B12] Celander DW, Cech TR. 1991 Visualizing the higher order folding of a catalytic RNA molecule. Science 251(4992): 401–407. 1989074 10.1126/science.1989074

[msz034-B13] Cotruvo JA, Stubbe J. 2011 Class I ribonucleotide reductases: metallocofactor assembly and repair in vitro and in vivo. Annu Rev Biochem. 80(1): 733–767. 21456967 10.1146/annurev-biochem-061408-095817 PMC4703083

[msz034-B14] Fahnestock S, Neumann H, Shashoua V, Rich A. 1970 Ribosome-catalyzed ester formation. Biochemistry 9(12): 2477–2483. 4912484 10.1021/bi00814a013

[msz034-B15] Fox GE. 2010 Origin and evolution of the ribosome. Cold Spring Harb Perspect Biol. 2(9): a003483. 20534711 10.1101/cshperspect.a003483 PMC2926754

[msz034-B16] Hsiao C, Chou IC, Okafor CD, Bowman JC, O’Neill EB, Athavale SS, Petrov AS, Hud NV, Wartell RM, Harvey SC, et al 2013 RNA with iron(II) as a cofactor catalyses electron transfer. Nat Chem. 5(6): 525–528. 23695635 10.1038/nchem.1649

[msz034-B17] Hsiao C, Lenz TK, Peters JK, Fang PY, Schneider DM, Anderson EJ, Preeprem T, Bowman JC, O’Neill EB, Lie L, et al 2013 Molecular paleontology: a biochemical model of the ancestral ribosome. Nucleic Acids Res. 41(5): 3373–3385. 23355613 10.1093/nar/gkt023 PMC3597689

[msz034-B18] Hsiao C, Mohan S, Kalahar BK, Williams LD. 2009 Peeling the onion: ribosomes are ancient molecular fossils. Mol Biol Evol. 26(11): 2415–2425. 19628620 10.1093/molbev/msp163

[msz034-B19] Hsiao C, Tannenbaum M, VanDeusen H, Hershkovitz E, Perng G, Tannenbaum A, Williams LD. 2008 Complexes of nucleic acids with group I and II cations. In: Hud N, editor. Nucleic acid metal ion interactions. London: The Royal Society of Chemistry p. 1–35.

[msz034-B20] Hsiao C, Williams LD. 2009 A recurrent magnesium-binding motif provides a framework for the ribosomal peptidyl transferase center. Nucleic Acids Res. 37(10): 3134–3142. 19279186 10.1093/nar/gkp119 PMC2691814

[msz034-B21] Job P. 1928 Studies on the formation of complex minerals in solution and on their stability. Ann Chim Fr. 9:113–203.

[msz034-B22] Johnson JE, Webb SM, Ma C, Fischer WW. 2016 Manganese mineralogy and diagenesis in the sedimentary rock record. Geochim Cosmochim Acta 173:210–231.

[msz034-B23] Klein DJ, Schmeing TM, Moore PB, Steitz TA. 2001 The kink-turn: a new RNA secondary structure motif. EMBO J. 20(15): 4214–4221. 11483524 10.1093/emboj/20.15.4214 PMC149158

[msz034-B24] Krupkin M, Matzov D, Tang H, Metz M, Kalaora R, Belousoff MJ, Zimmerman E, Bashan A, Yonath A. 2011 A vestige of a prebiotic bonding machine is functioning within the contemporary ribosome. Philos Trans R Soc Lond B Biol Sci. 366(1580): 2972–2978. 21930590 10.1098/rstb.2011.0146 PMC3158926

[msz034-B25] Lehman IR. 1974 DNA ligase: structure, mechanism, and function. Science 186(4166): 790–797. 4377758 10.1126/science.186.4166.790

[msz034-B26] Noller HF. 2004 The driving force for molecular evolution of translation. RNA 10(12): 1833–1837. 15547132 10.1261/rna.7142404 PMC1370670

[msz034-B27] Ohtomo Y, Kakegawa T, Ishida A, Nagase T, Rosing MT. 2014 Evidence for biogenic graphite in early Archaean Isua metasedimentary rocks. Nat Geosci. 7(1): 25–28.

[msz034-B28] Petrov AS, Bernier CR, Gulen B, Waterbury CC, Hershkovits E, Hsiao C, Harvey SC, Hud NV, Fox GE, Wartell RM, et al 2014 Secondary structures of rRNAs from all three domains of life. PLoS One 9(2): e88222. 24505437 10.1371/journal.pone.0088222 PMC3914948

[msz034-B29] Petrov AS, Bernier CR, Hsiao C, Norris AM, Kovacs NA, Waterbury CC, Stepanov VG, Harvey SC, Fox GE, Wartell RM, et al 2014 Evolution of the ribosome at atomic resolution. Proc Natl Acad Sci U S A. 111(28): 10251–10256. 24982194 10.1073/pnas.1407205111 PMC4104869

[msz034-B30] Petrov AS, Gulen B, Norris AM, Kovacs NA, Bernier CR, Lanier KA, Fox GE, Harvey SC, Wartell RM, Hud NV, et al 2015 History of the ribosome and the origin of translation. Proc Natl Acad Sci U S A. 112(50): 15396–15401. 26621738 10.1073/pnas.1509761112 PMC4687566

[msz034-B31] Riordan JF. 1977 The role of metals in enzyme activity. Ann Clin Lab Sci. 7(2): 119–129. 192123

[msz034-B32] Schopf JW, Kudryavtsev AB, Agresti DG, Wdowiak TJ, Czaja AD. 2002 Laser-Raman imagery of Earth’s earliest fossils. Nature 416(6876): 73–76. 11882894 10.1038/416073a

[msz034-B33] Selmer M, Dunham CM, Murphy FV, Weixlbaumer A, Petry S, Kelley AC, Weir JR, Ramakrishnan V. 2006 Structure of the 70S ribosome complexed with mRNA and tRNA. Science 313(5795): 1935–1942. 16959973 10.1126/science.1131127

[msz034-B34] Sievers A, Beringer M, Rodnina MV, Wolfenden R. 2004 The ribosome as an entropy trap. Proc Natl Acad Sci U S A. 101(21): 7897–7901. 15141076 10.1073/pnas.0402488101 PMC419528

[msz034-B35] Williamson JR, Raghuraman MK, Cech TR. 1989 Monovalent cation-induced structure of telomeric DNA: the G-quartet model. Cell 59(5): 871–880. 2590943 10.1016/0092-8674(89)90610-7

