# Supplementary material for: Prebiotic Iron Originates the Peptidyl Transfer Origin
Source: Mol Biol Evol. 2019 Feb 19;36(5):999–1007. doi: 10.1093/molbev/msz034 (PMC6502087; doi:10.1093/molbev/msz034)
Supplement: Supplementary Data [file msz034_supp.zip › revised_SI_submit.pdf]

Supporting information.

Title: Prebiotic iron originates the peptidyl transfer origin

Authors: Shin-Yi Lin, Ying-Chi Wang and Chiaolong Hsiao*

Affiliations: Institute of Biochemical Sciences, National Taiwan University, Taipei 10617, Taiwan

Corresponding author: Chiaolong Hsiao, Institute of Biochemical Sciences, National Taiwan University, Taipei 10617, Taiwan
Email: chiaolong@ntu.edu.tw

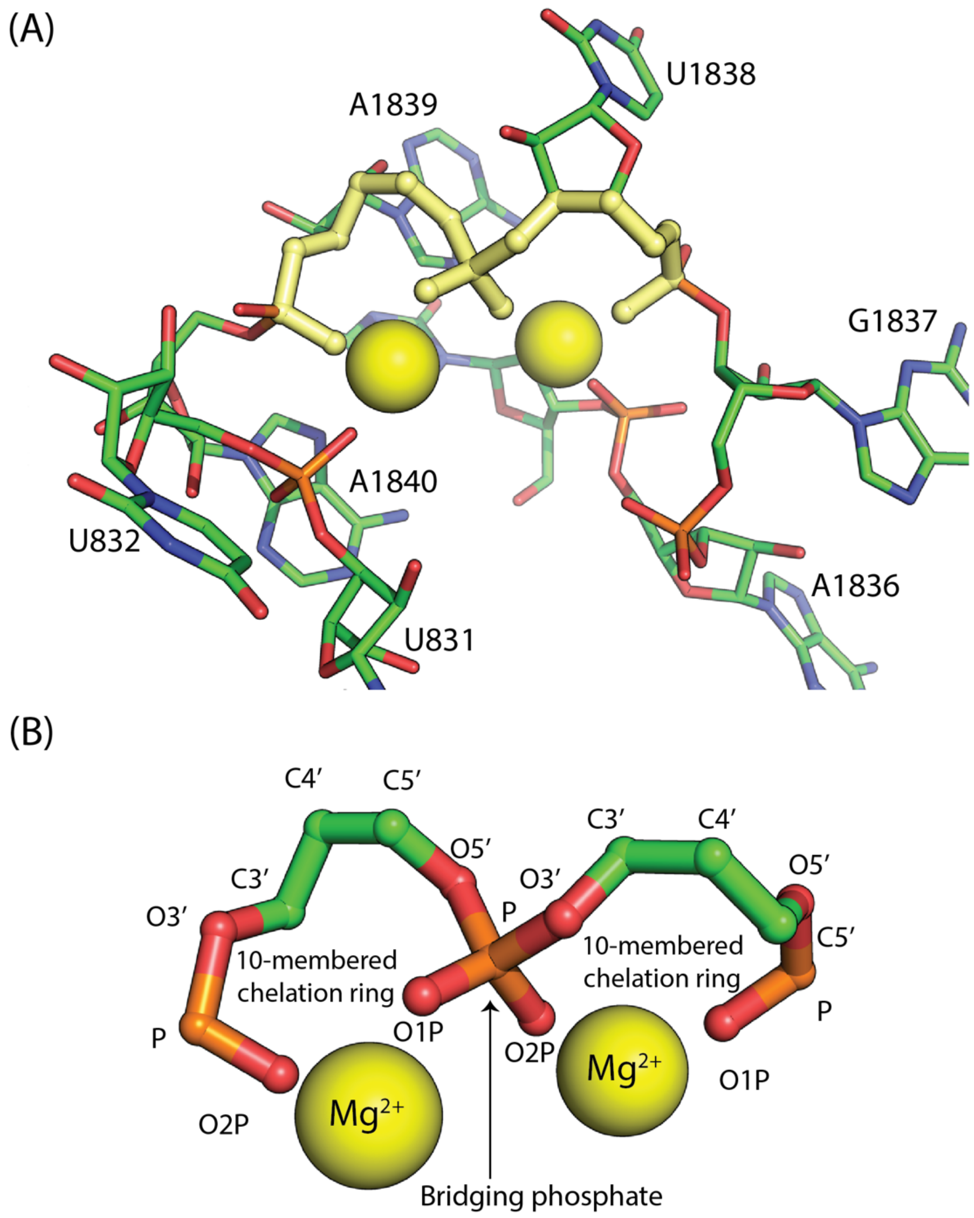

Figure S1. One of the four Mg^{2+}-μc's (Hsiao and Williams 2009) of *H. marismortui* LSU (PDB entry 1JJ2) shown at atomic level representations. (A) The Mg^{2+}-μc with bases and riboses included. The structural character of Mg^{2+}-μc is highlighted in yellow. (B) A close-up view of the Mg^{2+}-μc with bases and riboses omitted. Common features of the Mg^{2+}-μc include two idiosyncratic Mg^{2+} ions (yellow spheres), bridging phosphate and 10-membered chelation ring(s).

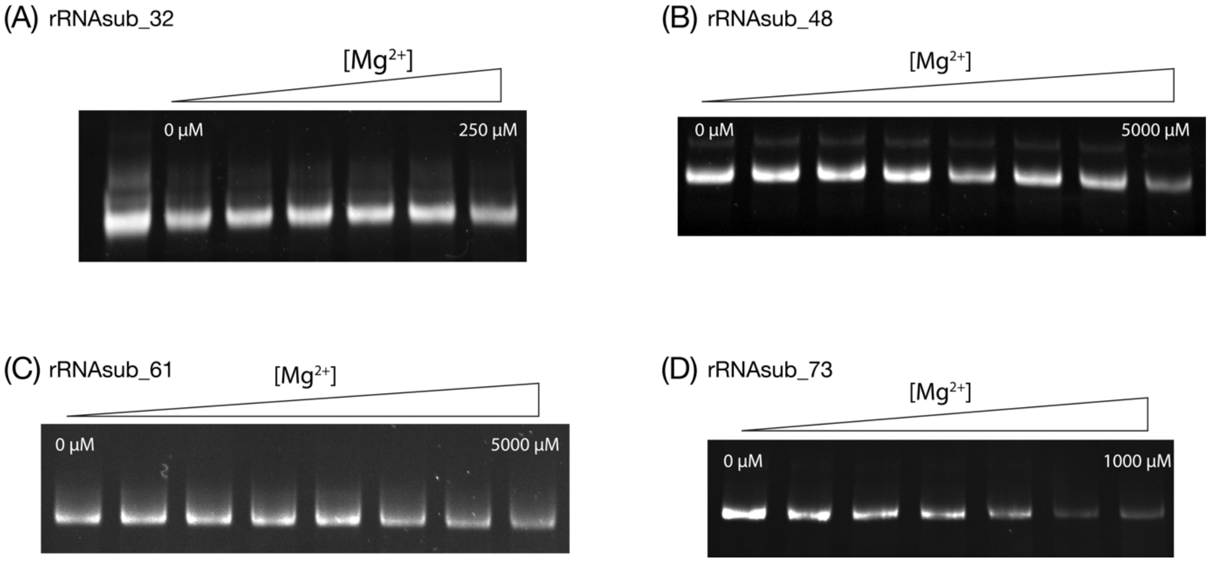

Figure S2. The gel mobility of rRNAsubs with Mg^{2+}. (A) The rRNAsub_32 with Mg^{2+} suggests an induction of folding. The first left lane was the fresh prepared transcript that exhibits multiple conformations of the RNA. Shown is the rRNAsub_32 annealed with varying [Mg^{2+}], resolved on a 11.5% native acrylamide gel. The Mg^{2+} concentration in Lane 2 is 0 µM, Lane 3: 12.5, Lane 4: 2.5, Lane 5: 50, Lane 6: 100 and Lane 7: 250. (B) The rRNAsub_48 with Mg^{2+} suggests an induction of folding. Shown is the rRNAsub_48 annealed with varying [Mg^{2+}], resolved on a 10.5% native acrylamide gel. The Mg^{2+} concentration in Lane 1 is 0 µM, Lane 2: 25, Lane 3: 50, Lane 4: 100, Lane 5: 250, Lane 6: 500, Lane 7: 1000 and Lane 8: 5000. (C) The rRNAsub_61 with Mg^{2+} suggests an induction of folding. Shown is the rRNAsub_61 annealed with varying Mg^{2+} concentration solution, resolved on a 11% native acrylamide gel. The Mg^{2+} concentration in Lane 1 is 0 µM, Lane 2: 25, Lane 3: 50, Lane 4: 100, Lane 5: 250, Lane 6: 500, Lane 7: 1000 and Lane 8: 5000. (D) The rRNAsub_73 with Mg^{2+} suggests an induction of folding. Shown is the rRNAsub_73 annealed with varying Mg^{2+} concentration solution, resolved on a 7% native acrylamide gel. The Mg^{2+} concentration in Lane 1 is 0 µM, Lane 2: 25, Lane 3: 50, Lane 4: 100, Lane 5: 250, Lane 6: 500 and Lane 7: 1000. All the RNA samples were first pre-treated with cation

exchange resin (Chelex 100 Resin, Bio-RAD) and then prepared and annealed in 50mM Tris-Cl, pH 8.0 with varying concentration of Mg^{2+}solution.

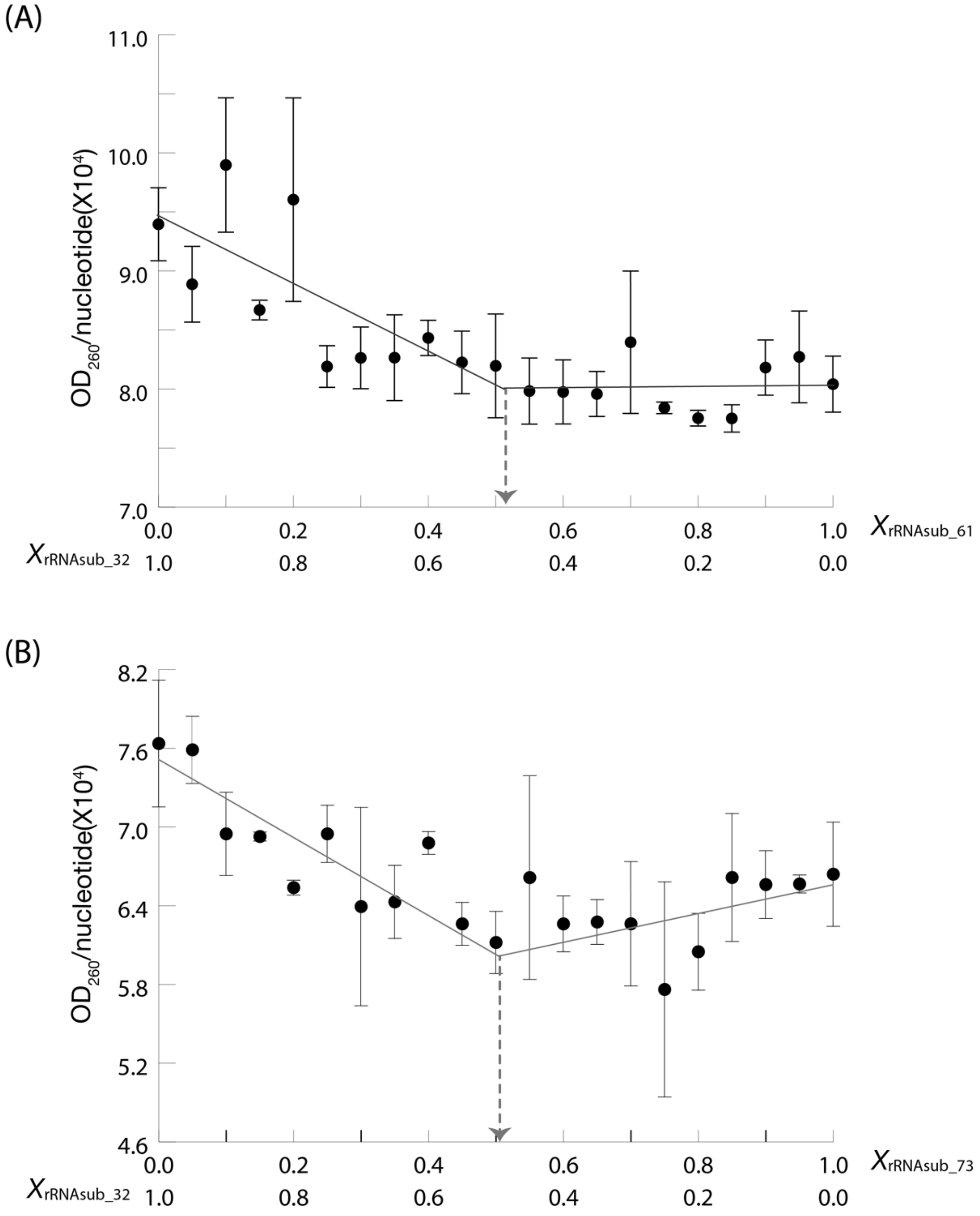

Figure S3. The continuous variation experiment on the rRNAsubs in the presence of Fe^{2+}. Shown is the continuous variation analysis (Job 1928; Cantor and Schimmel 1984) on (A) the rRNAsub_61 and rRNAsub_32, and (B) the rRNAsub_73 and rRNAsub_32. In the plots, values on horizontal axis denote mole fraction of the two rRNAsubs in each sample. The two rRNAsubs were mixed and held constant at 0.2 μM and at 0.1 μM for [rRNAsub_73+rRNAsub_32], while the mole fractions of the two components were varied from 0.0 to 1.0. The samples were prepared in 50 mM Tris-Cl buffer, pH 8 and 32 μM Fe^{2+}

solution. The discontinuity at equivalent mole fractions of the two rRNAsubs indicates a complex with ratio of stoichiometry.

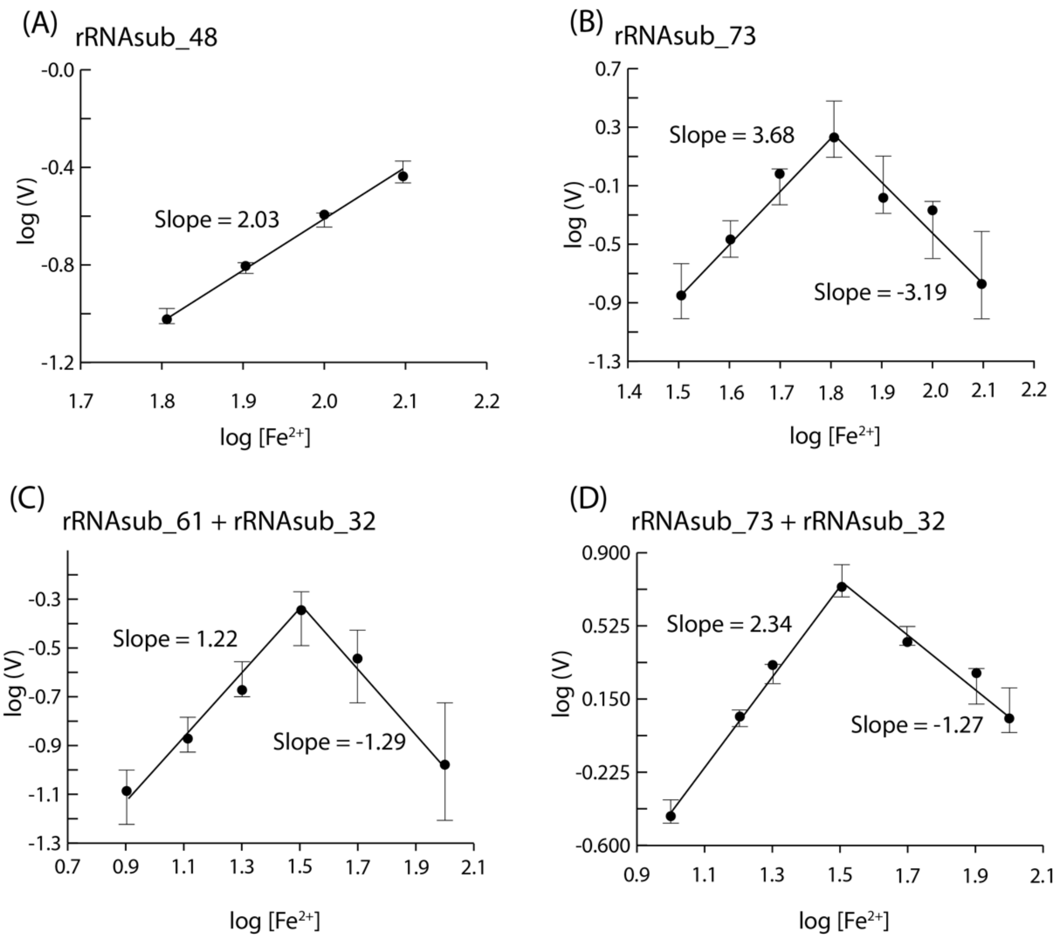

Figure S4. The iron dependence analysis. Shown is the plot of natural logarithm of Fe^{2+} concentration versus natural logarithm of initial rate of electron transfer performed by (A) rRNAsub_48, (B) rRNAsub_73, (C) rRNAsub_61 and rRNAsub_32 and (D) rRNAsub_73 and rRNAsub_32. The slopes were obtained by linear regression fitting models. A positive slope indicates the number of Fe^{2+} bound to the specific di-nuclear metal binding site of the RNA, while a negative slope suggests a deformation of the Fe^{2+}-μc in the incremental Fe^{2+}.

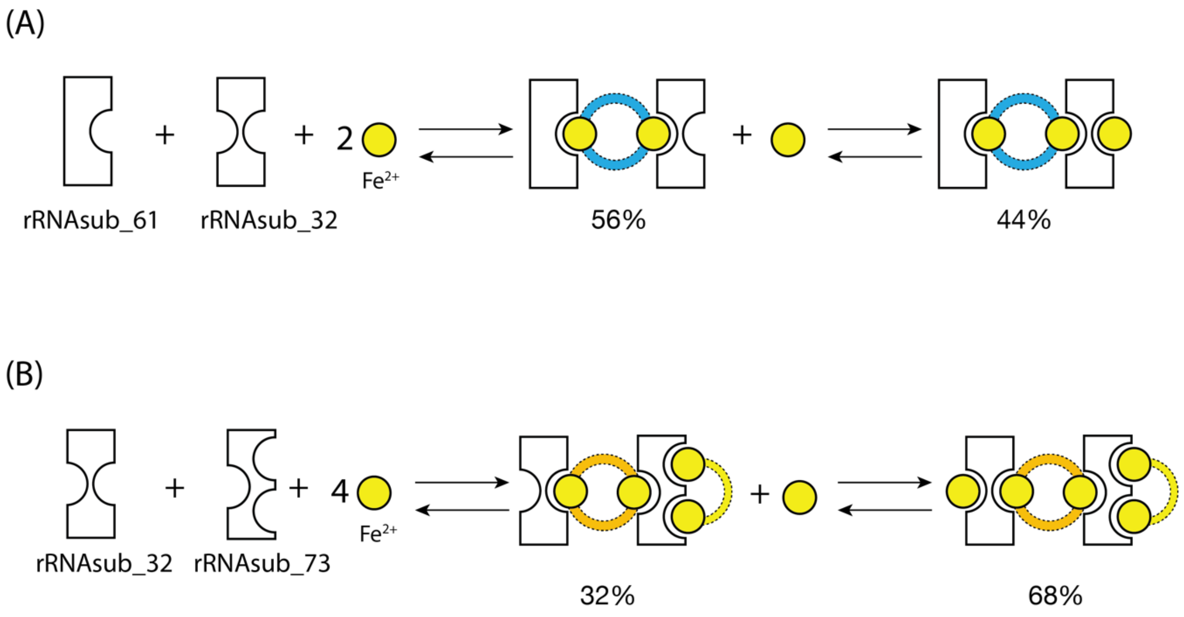

Figure S5. The Fe^{2+}-μc assembly. The iron dependency experimental results (Figure S3), combined with the continuous variation analysis (Figure 3), allow us to deduce that in panel (A) two idiosyncratic Fe^{2+} cations bound to the [rRNAsub_61 + rRNAsub_32] assembly to form a di-nuclear Fe^{2+} entity is 56% verses an auxiliary Fe^{2+} continuously bound to that complex is 44%, and in panel (B) the case of [rRNAsub_73 + rRNAsub_32] assembly, a pair of idiosyncratic Fe^{2+} cations are bound to form a two di-nuclear Fe^{2+}-rRNA complex is 32% verses an auxiliary Fe^{2+} continuously bound to that complex is 68% (see Appendix I for details in calculations).

Table S1. The gene sequence of the rRNAsubs used in the current experiments.

Name	Residue number[a] (5'to 3')	Size (bp)	Sequence[b]: from 5' to 3'
rRNAsub_73	(2886-2127)-TL-(2266-2658)	445	AAGCTTTAATACGACTCACTATAGGCCCAGGGGGAAGCG AAGACCCTATGGAGCTTTACTGCAGGCTGGGGTAACCCA GCCGGGCAGTTTGACTGGGGCGGTACGCGCTCGAAAAG ATATCGAGCGCGCCCTATGGCTATCTCAGCCGGGACAGA GACCCGGCGAAGAGTGCAAGAGCAAAAGATAGCTTGAC AGTGTTCTTCCCAACGAGGAACGCTGACGCGAAAGCGT GGTCTAGCGAACCAATTAGCCTGCTTGATGCGGGCAATT GATGACAGAAAAGCTACCCTAGGGATAACAGAGTCGTC ACTCGCAAGAGCACATATCGACCGAGTGGCTTGCTACCT CGATGTCGGTTCCCTCCATCCTGCCCGTGCAGAAGCGGG CAAGGGTGAGGTTGTTCGCCTATTAAAGGAGGTCGTGA GCTGGGTTTAGACCGTCGTGAGACAGGTCGGCTGCTATC TACTGGGGGATCC
rRNAsub_61	(1734-1887)-TL-(2015-2044)	196	AAGCTTTAATACGACTCACTATAGGCCAACGTTAGGGA ATTCGGCAAGTTAGTCCCGTACCTTCGGAAGAAGGGATG CCTGCTCCGGAACGGAGCAG GTCGCAGTGACTCGGAAGCTCGGACTGTCTAGTAACAA CATAGGTGACCGCAAATCCGCAAGGACTCGTACGGTCA CTGAATGGGGTAACCCATTAACCAGAGCTTCACTGTCCC AACGTTG GGATCC
rRNAsub_48	(1401-1520)-TL-(1666-1719)	184	AAGCTTTAATACGACTCACTATAGG GGCCTAATGGATAAGGGTTCCTCAGCACTGCTGATCAGC TGAGGGTTAGCCGGTCCTAAGTCATACCGCAACTCGACT ATGACGAAATGGGAAACGGGTTAATATTCCCGTGCCACT ATGGGGGTAACCCCATAGTGTCCGTACCGAGAACCGAC ACAGGTGTCCATGGCGGCGAAAGCCAAGGCC GGATCC
rRNAsub_32	(770-891)	122	AAGCTTTAATACGACTCACTATAGGCGGACGATCTACGC ATGGACAAGATGAAGCGTGCCGAAAGGCACGTGGAAGT CTGTTAGAGTTGGTGTCCTACAATACCCTCTCGTGATCTA TGTGTAGGGGTGAAAGGCCCATCGAGTCCGGGATCC

[a] Numbers in the parenthesis are continuous residue numbers of the *Haloarcula marismortui* 23S rRNA sequence. The TL stands for tetraloop with a sequence GGGGTAACCC to connect the two fragments.

[b] The T7 promoter gene is highlighted green, the TL is highlighted cyan with red letters and the two restriction sites are HindIII at 5'- and BamHI at 3'-end, highlighted gray.

Table S2. The initial rate (v_0) of reaction for rRNAsubs in the presence of Fe^{2+} and Mn^{2+}.

**rRNAsubs**	v_0^{Fe+Mn} **(μM/min.) [Fe^{2+} and Mn^{2+}]**	v_0^{Fe} **(μM/min.) [Fe^{2+}]**	**Fold of increment. (v_0^{Fe+Mn} / v_0^{Fe})**
rRNAsub_73	1.06	0.82	1.3
rRNAsub_61 + rRNAsub_32	7.59	1.50	5.1
rRNAsub_73 + rRNAsub_32	10.84	5.21	2.1
rRNAsbu_48	4.06	0.46	8.8

Appendix I.

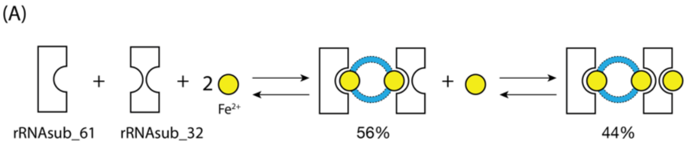

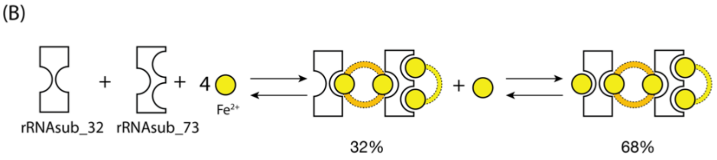

(A) [rRNAsub_61 + rRNAsub_32] assembly with Fe^{2+}.

We obtained a slope of 1.22 for [rRNAsub_61 + rRNAsub_32] in iron dependence analysis (Figure S3C). From continuous variation experiments, we obtained a 1:1 stoichiometric complex of [rRNAsub_61 + rRNAsub_32] (Figure S2A), and a 1:1 stoichiometric complex of [rRNAsub_61 + rRNAsub_32] and Fe^{2+} (Figure 3E). If [$(rRNAsub\_61)_1(rRNAsub\_32)_1(Fe^{2+})_2$] complex is formed 100%, ideally, the slope in the iron dependence would be 1. However, we obtained the slope of 1.22, which implies that maybe other complex is formed in the equilibrium. We assume the other complex is [$(rRNAsub\_61)_1(rRNAsub\_32)_1(Fe^{2+})_3$]. Thus, in the equilibrium, there would be X% of [$(rRNAsub\_61)_1(rRNAsub\_32)_1 (Fe^{2+})_2$] and Y% of [$(rRNAsub\_61)_1(rRNAsub\_32)_1(Fe^{2+})_3$].

Therefore,

$X + Y = 1$

$(2/2)*X + (3/2)*Y = 1.22$

Solve for X and Y, we got 0.56 for X and 0.44 for Y.

(B) [rRNAsub_73 + rRNAsub_32] assembly with Fe^{2+}.

The same calculation shown above can be applied here to obtain, in the equilibriums, there are 32% of [$(rRNAsub\_32)_1(rRNAsub\_73)_1 (Fe^{2+})_4$] and 68% of [$rRNAsub\_32)_1(rRNAsub\_73)_1 (Fe^{2+})_5$] complexes.

References.

Cantor C, Schimmel P. 1984. Biophysical Chemistry (I-III). New York: Academic Press.

Hsiao C, Williams LD. 2009. A recurrent magnesium-binding motif provides a framework for the ribosomal peptidyl transferase center. Nucleic Acids Res 37:3134-3142.

Job P. 1928. Studies on the formation of complex minerals in solution and on their stability. Annales De Chimie France 9:113-203.
